# Gaining masculine power through guns? The impact of masculinity threat on attitudes toward guns

**DOI:** 10.3389/fpsyg.2024.1296261

**Published:** 2024-02-15

**Authors:** Sven Kachel, Tibor Bloch, Jennifer K. Bosson, Lea L. Lorenz, Melanie C. Steffens

**Affiliations:** ^1^Department of Social, Environmental, and Economic Psychology, University of Kaiserslautern-Landau, Landau, Germany; ^2^Department of Languages, University of Helsinki, Helsinki, Finland; ^3^Department of Psychology, University of South Florida, Tampa, FL, United States

**Keywords:** masculinity, identity threat, gender role, precarious manhood, toxic masculinity, firearms, guns

## Abstract

Gun violence is a serious problem in the United States and elsewhere and more so among men than women. We conducted an experiment to examine if men whose masculinity was threatened are more attracted to guns than non-threatened men, presumably to compensate for the threat. After completing a gender knowledge test, men (*N* = 168) randomly received either false masculinity threatening (experimental condition) or masculinity affirming (control condition) feedback. Subsequently, we measured men’s attitudes toward guns and their choice of a gun-range voucher. Men whose masculinity was threatened (vs. affirmed) showed more positive attitudes toward guns and were more likely to choose the voucher. Both effects were statistically significant when the whole sample was analyzed and when very strict exclusion criteria were applied. However, when data exclusions were based on a suspicion check, effects were statistically significant only when a covariate was included (i.e., social dominance orientation, patriotism, or experience with guns). We discuss reasons for this mixed evidence, including the possibility that suspicion regarding the masculinity feedback could itself be a compensatory reaction to threat.

## Introduction

About 30% of all American citizens own a gun (Parker et al., 2017; for trends on United States gun ownership since 2007, see Saad, 2020). According to the Gun Violence Archive (2022) during the year 2021 more than 45,000 persons died by means of a firearm in the United States overall, and more than 40,500 persons were injured by firearms. These numbers illustrate that the topic is highly relevant for public health and safety, police work, justice, as well as politics, economy, and family relations, to name a few. At the same time, controlling firearms is a policy issue provoking strong controversies, and the public discussion surrounding this topic is characterized by often irreconcilable opposing positions (Miller, 2019). Consequently, there has been a call for more research on gun policies and safety (Winker et al., 2016).

Only 19% of American women compared to 45% of American men own at least one gun (Saad, 2020). Thus, men appear to be more attracted to guns than women. The central aim of the present work is to find out more about the relationship between masculinity threats and men’s attraction to guns. A masculinity threat can be anything that is perceived as a threat to one’s manhood, including direct and interpersonal feedback or a subtle suggestion that one is not meeting expectations regarding men (Borgogna et al., 2022). For this purpose, we present an experiment examining the effect of a masculinity threat on men’s attraction to guns. We propose that men, when threatened with respect to their masculinity, feel the need to compensate for this and that one compensatory mechanism can consist of an overly masculine self-presentation. Since guns symbolize masculine power and strength (Stroud, 2012), men should be attracted more to these objects after having experienced a masculinity threat.

Precarious manhood theory states that the social status of being a man is characterized by more instability and insecurities, compared to the social status of being a woman. Therefore, masculine status is more difficult to reach and maintain, plus it can be threatened and damaged or lost more easily (Vandello and Bosson, 2013; but see Chrisler, 2013, for arguments why the social status of being a woman is also insecure). Precarious manhood theory rests on three central pillars. First, manhood as a social status requires that men fulfill certain behavioral conditions in addition to biological ones. Second, manhood status is relatively impermanent and men can easily lose their status in others’ eyes. Third, men must regularly prove their masculinity. When a man is confronted with any situation that challenges his masculinity, he tries to reaffirm and confirm his status as a man.

The fundamental assertion stated by this theory, that the social status as a man is relatively unstable, is supported by another research group (Willer et al., 2013), using a slightly different reasoning: On a societal level, the descriptive and injunctive norms concerning how men are expected to be and behave dictate a relatively limited pool of socially accepted traits and behaviors for men as compared to women (also see Diekman and Eagly, 2000). Consequently, manhood status is threatened more easily than womanhood status: Men who do not uphold this narrow scope of masculinity standards can lose their status rapidly.

According to the masculine overcompensation thesis (Willer et al., 2013), when men feel insecurity about their masculinity, they overcompensate by exaggerating manly behavior. Exaggerated in this context signifies that men display behaviors in an excessive manner that are seen as manly in the eyes of the surrounding community and/or in their own eyes. Overcompensation serves to reaffirm and confirm the identity in question (for comparable reasoning, see Gollwitzer et al., 1982). What could such an overcompensation look like?

Expressing positive attitudes toward guns could be one direct route by which men can compensate for threatened masculinity (O’Neill, 2007; Stroud, 2012; Carlson, 2015). For instance, the more men experienced stress for not being able to comply with the societal norms for masculinity, the more attraction to guns they reported (Scaptura and Boyle, 2022; also see Ray et al., 2021). Other evidence shows that in areas where larger numbers of households contain unemployed husbands married to employed wives, firearm background check inquiries increase (Cassino and Besen-Cassino, 2020). This perhaps indicates that men who feel threatened by their inability to play the traditional “breadwinner” role compensate, in part, by purchasing guns, thus stressing their family-protector role.

Similar studies suggest that several individual differences could moderate the relationship between masculinity threat and attraction to guns. White gun owners reported feeling more empowered through guns when they experienced greater economic precarity (Mencken and Froese, 2019). This tendency was strongest among men who more strongly endorsed masculine role attitudes (Warner et al., 2022). McDermott et al. (2021) found that owning firearms is not only linked to being White, politically conservative, and a man, but also to adhering to masculine role norms (including norms of violence and power over women). In addition to these masculine roles and identification (e.g., Maass et al., 2003), in United States men, masculine honor ideology could also be an important moderator variable (e.g., Barnes et al., 2012; Stratmoen et al., 2018).

Gun attitudes are embedded in participants’ more general belief systems and thus should be related to several other constructs. First, gun ownership goes along with more positive attitudes toward guns (Tenhundfeld et al., 2020). More generally, gun experience, whether positive or negative, affects attitudes toward guns (Shapira et al., 2022). Second, because guns are emblematic of aggression (for a review, see Benjamin and Bushman, 2016) and United States -American hegemonic masculinity includes being aggressive (e.g., DiMuccio and Knowles, 2023), we included several relevant constructs that are related to aggression across various domains. These include not only general aggressiveness, but also patriotism (Mencken and Froese, 2019). Additionally, we included two individual-difference characteristics that are considered powerful predictors across a wide range of social, political, and intergroup attitudes and behavior: social dominance orientation (SDO, e.g., Pratto et al., 1994), defined as the degree to which people approve of group-based hierarchies (Pratto et al., 2013), and right-wing authoritarianism, representing the degree to which people seek conformity, value order, and submit to authority (e.g., Jonathan, 2008; Bizumic and Duckitt, 2018; Austin and Jackson, 2019). For example, SDO covaries with attitudes supportive of weapon use and military actions (Pratto et al., 1994), and thus could account for some of the variance in people’s attraction to guns.

Taken together, we argue that men whose masculinity is threatened should be more attracted to guns than men whose masculinity is not threatened because feeling more attracted to guns can be a compensatory reaction restoring masculinity. Indeed, the first experimental evidence for this recently showed that masculinity-threatened men reported higher interest in owning specific firearms than non-threatened men (Borgogna et al., 2022).

### The present study

We propose that men, when threatened with respect to their masculinity, will compensate by reporting more attraction to guns. To this aim, we assigned male participants to a masculinity-threatening experimental versus a masculinity-affirming control condition. While conceptually replicating the recent experiment by Borgogna et al. (2022), our study extended theirs in several ways. First, ours was designed as an in-person experiment, whereas Borgogna et al.’s (2022) was an online study. Second, we measured participants’ general attitudes toward guns, while those authors measured specific attraction toward several displayed firearms. Third, we included a behavioral outcome, consisting of a (fake) voucher for shooting training at a local gun range. Participants could choose to take this voucher or not. This extension follows the frequent call to include “real” consequential behavior in social-psychological research instead of only measuring attitudes (for discussion, see, e.g., Bartos et al., 2014; Preuß et al., 2020). Fourth, instead of the fake personality profile that Borgogna et al. (2022) used to induce masculinity threat, our manipulation consisted of the often-used gender knowledge test (e.g., Lamarche et al., 2021). Finally, several moderators and covariates were included in our study (see Supplement for full list). In addition to political ideology, we assessed social dominance orientation (e.g., Pratto et al., 2013), patriotism, general aggressiveness (for both, see Barnes et al., 2012), as well as authoritarianism (e.g., Bizumic and Duckitt, 2018). Beyond that, several potential moderator variables concerning individual differences in conceptions and identification with masculinity were explored (also see McDermott et al., 2021; Warner et al., 2022) that did not yield expected findings and are therefore reported in the Supplement: masculine honor ideology (e.g., Barnes et al., 2012), masculine gender-role self-concept (Kachel et al., 2016), and conformity to masculine norms (e.g., Levant et al., 2020).

The study was pre-registered. Preregistration, data, and supplementary information are available online.^1^

### Hypotheses

*H1a*: Men whose masculinity is threatened will exhibit more positive attitudes toward guns than men whose masculinity is not threatened.

*H1b*: Men whose masculinity is threatened will be more likely to choose a free voucher for a gun range than men whose masculinity is not threatened.

## Method

### Participants

We conducted an *a priori* sample size calculation using G*Power 3.1.9.7 (Faul et al., 2007). To detect at least medium-sized effects (*d* = 0.5) using a one-tailed independent samples *t*-test (α = 0.05, 1 – β = 0.90), 140 participants were needed. Considering possible attrition, we planned to recruit 174 participants. To be eligible, participants had to be: male, between 18 and 59 years of age, an American citizen or permanent resident, and fluent in English.

The study was approved by the Institutional Review Board of the University of South Florida (USF). Participants were recruited in three ways. First, student members of the USF Psychology Department participant pool signed up online in exchange for course credit. Second, flyers offering 10 $ for participation were posted on the USF campus. Third, because the planned sample size was not obtained, potential participants (e.g., men sitting on a park bench) were additionally approached in public spaces off-campus and invited to complete the study in exchange for 10 $ if they were residents of Tampa or Hillsborough County. Participants recruited on the USF campus (*n* = 77) participated in a laboratory room in the Psychology building; those recruited off-campus participated in the location where they were recruited, on portable tablet computers (*n* = 91). The pattern of findings reported below remains if setting is (additionally) controlled for.

Having reached a sample size of 168 participants, data collection ended on May 5th, 2022. A sensitivity analysis showed that effects of *d* ≥ 0.45 could be detected with this sample size. Participants’ average age was 21.5 years (*SD* = 5.9, range 18–57). Most participants were White/Caucasian (67.3%), with most others identifying as African-American (6.5%), Asian-American (7.1%), or Middle-Eastern-North African (3%). The pattern of reported findings remains if “White/Caucasian vs. other” is (additionally) controlled for. The majority was from Florida (67.9%), came originally from a suburban community (57.7%), and had not completed any level of education beyond high school yet (76.2%). Almost all were United States citizens (99.4%) and 86.9% stated English was their first language.

On average, political ideology (“When it comes to politics in general, I am…”; 1 = *very liberal* to 7 = *very conservative*) was 3.69 (*SD* = 2.51; Democrats: 33.9%, Republicans: 30.4%, Independents: 25%). Most participants did not own a gun personally (90.5%). The pattern of reported findings remains if gun ownership is (additionally) controlled for. Experience with guns (i.e., “How much personal experience do you have with guns, or with shooting guns?”; 1 = *none at all* to 9 = *a lot*) was relatively low, *M* = 3.30, *SD* = 2.51. Gun experience was used as a covariate in some analyses below.

### Materials

#### Attitudes toward guns

The Gun Attitude Scale (GAS; Tenhundfeld et al., 2020) consists of nine items loading on one single factor. Responses are made on a scale ranging from 1 (*disagree strongly*) to 5 (*agree strongly*). Example items are: “I would personally feel safer by owning a gun,” and “I support the right to own a firearm.” We added a tenth item assessing a belief about guns pertaining to men’s stereotypically prescribed protector role (“I could be a better protector for my family if I had a gun”). An exploratory factor analysis confirmed the one-factor structure. The internal consistency of the 10-item scale was excellent (Cronbach’s α = 0.93). Responses were averaged, with higher scores indicating more positive attitudes toward guns.

#### Shooting range voucher

We created a fake voucher for a local shooting range offering three options: A free beginner firearms training (1 h), a free firearm experience for those with prior shooting experience (1 h), or no voucher. Responses were coded as taking a shooting range voucher or not (see below for cover story).

#### Potential covariates

Four potential covariates were included (in addition to gun experience described above). These were social dominance orientation (Short Social Dominance Orientation scale; Pratto et al., 2013; α = 0.72), patriotism (3 items by Barnes et al., 2012, α = 0.86), general aggressiveness (also following Barnes et al., 2012; α = 0.84), and authoritarianism (Very Short Authoritarianism scale; Bizumic and Duckitt, 2018, α = 0.60). Aggressiveness and authoritarianism had no significant effects and will not be mentioned further.

### Procedure

One of six experimenters introduced each participant to the study. Participants worked through the survey on the PC (in the lab) or laptop/tablet computer (in public places), depending on where they were recruited. At the end, the investigator conducted a suspicion check, debriefed, and compensated the participant. The procedure took on average 30–40 min.

The survey was created using Qualtrics.^2^ Participants were told that the study goal was to validate and norm social psychological scales. After that, the above-presented scales were presented to participants in two different (counterbalanced) orders. In one order, potential moderators and covariates were presented at the beginning, and in the other order, after the gun attitude scale (the order of moderators and covariates always was: masculine honor ideology, authoritarianism, conformity to masculine norms, social dominance orientation, masculine gender-role self-concept, feminine gender-role self-concept). As we found no significant differences of orders in the dependent measures, we collapsed across order when analyzing data.

Participants were then randomly assigned to one of two conditions (masculinity threat vs. control) that were based on Vandello et al. (2008), Study 4, see also Rudman and Fairchild (2004), Experiment 3. The threat was administered via false feedback about performance on a “gender knowledge test” (see also Weaver and Vescio, 2015; Berke et al., 2017; Stanaland and Gaither, 2021; Valved et al., 2021). The alleged test consisted of 32 items, measuring knowledge about stereotypically masculine and feminine topics (e.g., sports, home repair vs. childcare, fashion). Each item could be answered by choosing one of two response options (e.g., “The first people to use primitive flamethrowers in battle were [Greeks vs. Turks]”). As the items were difficult, false feedback was presumably believable to the participants.

After having completed the test, half of the participants received false masculinity-threatening feedback, consisting of a statement that their own score was close to the average U.S. American woman. Participants in the control condition received the feedback that their score was close to the average United States American man, which thus affirmed their masculinity. Both feedbacks were accompanied by a graph illustrating the scores (adapted from Vescio et al., 2021).

After reading their feedback, participants completed the gun attitudes scale. A total of three attention check items were interspersed between items of this scale and the covariates and moderators. Following the preregistration, no participant was excluded for non-attention because they all responded correctly to at least two of the attention checks (*n* = 9 made one error). Next, demographic information was collected. Participants then learned that the study was over, but to thank them for participating, they were being offered a free voucher from one of several local businesses. Participants watched a brief video mentioning four local businesses (one of which was a shooting range), and then learned that they had been randomly selected to receive a voucher from the shooting range. They could select to accept the voucher (and choose either the beginner or experienced shooter option) or decline the voucher. At the end of the survey participants were asked the questions concerning gun ownership and experience with guns (see Participants).

### Robustness checks

A comprehensive in-person suspicion check was conducted with each participant, focusing on the false feedback after the gender knowledge test plus the false shooting range voucher. It consisted of a set of standardized questions (see study script, Supplement) that the experimenter used to rate each participant’s degree of suspicion (scale 1–5). We preregistered (too vaguely) that participants “who failed the suspicion check” would be excluded. If we applied the criterion of suspicion rated 4–5 (reasoning that this indicated affirming some level of suspicion), this would result in 27 participants (16.1%) being excluded.

In hindsight, this procedure appeared questionable in several respects. First, as we will discuss (see Discussion), questioning the validity of the feedback is itself a potential means of restoring threatened masculinity. Thus, men may have reported suspicion defensively. Second, we suspect there was an experimenter bias because the probability of being excluded depended on the experimenter (exclusion rate varied from 0 to 17%), suggesting that experimenters had different decision thresholds. Finally, the probability to be excluded depended on condition, χ^2^(1) = 3.08, one-tailed *p* = 0.039. Suspicion was more likely in the masculinity-threat than control condition. Thus, random assignment of participants to conditions was violated for this sample (i.e., an individual excluded from the masculinity-threat condition would have been retained had they been in the control condition).

For these reasons, we decided on using extreme inclusion/exclusion criteria as robustness checks. In detail, we used (a) a maximum sample (i.e., excluding no one) corresponding to a very lenient criterion, (b) a no-suspicion sample (i.e., excluding participants who had been rated suspicious), and (c) a minimum sample (i.e., excluding all participants whose data were potentially of bad quality; e.g., they had taken social psychology or indicated they had consumed drugs or alcohol before the experiment) corresponding to a very strict criterion. This practice of using both a very lenient and a very strict exclusion criterion is common, for instance, in memory psychology when scoring free-recall responses in order to test whether findings depend on the scoring criterion (e.g., Steffens et al., 2003). Taken together, we report findings using (a) the whole sample (*N* = 168), (b) the “no-suspicion sample” (*n* = 141), and (c) a minimal sample (*n* = 103). In addition, supplementary robustness checks are reported controlling for covariates.

## Results

Data were analyzed using IBM SPSS Statistics Version 27.0.1.0. Assumptions with respect to all statistical analyses were fulfilled or, if not, robust methods were applied instead. One-tailed tests are reported for all relationships where theory allows directional hypotheses (Meiser, 2011).

### Hypothesis testing

#### Attitudes toward guns

As shown in Table 1, descriptively attitudes toward guns were more positive in the masculinity-threat compared to the control condition in all three samples. This difference was statistically significant in the maximum sample, supporting Hypothesis 1a, but not in the no-suspicion sample; in the minimal sample, *p* = 0.05.

**Table 1:** Average attitudes toward guns (with *SDs*) by condition and results of independent-samples *t*-tests for the maximum sample (*N* = 168), the no-suspicion sample (*N* = 141), and the minimum sample (*N* = 103).

	Condition			Statistics				95% CI (d)	
Sample	Threat	Control	Difference	*t*	*df*	*p* ^a^	*d*	LL	UL
Maximum	3.43 (0.95)	3.10 (1.09)	0.34	2.16	166	0.017	0.33	0.03	0.64
No suspicion	3.38 (0.99)	3.12 (1.11)	0.26	1.48	139	0.071	0.25	−0.08	0.58
Minimum	3.39 (0.86)	3.07 (1.11)	0.32	1.66	100	0.050	0.32	−0.07	0.71

^a^One-tailed.

#### Shooting range voucher

Using a χ^2^-test, we examined if voucher choice differed by masculinity threat manipulation. Table 2 shows the findings. Descriptively, in all three samples men were more likely to choose a shooting range voucher in the threat versus control condition. This difference was statistically significant in the maximum and the minimum sample, confirming Hypothesis 1b, but not in the no-suspicion sample.

**Table 2:** Percentages of participants taking vouchers by condition and results of χ^2^-tests in the maximum (*n* = 153), no-suspicion (*n* = 120), and minimum sample (*n* = 88).

Sample	Condition		Test statistics			
	Threat	Control	χ^2^	*df*	*p* ^a^	φ
Maximum	65.0	50.7	3.22	1	0.037	0.15
No suspicion	63.8	51.6	1.82	1	0.089	0.12
Minimum	67.5	50.0	2.74	1	0.049	0.18

^a^One-tailed. Ns are smaller than in Table 1 because of missing data. Additionally, eight more participants were excluded from the no-suspicion sample because they indicated suspicion about the vouchers only.

### Additional robustness checks including covariates

We explored potential covariates’ influence on the effect of condition on attitudes toward guns. Three covariates (social dominance orientation, patriotism, experience with guns) were substantially related with gun attitudes (see Table 3). As one would expect based on the gun-attitudes literature, participants held significantly more positive attitudes toward guns the higher their social dominance orientation, the higher their patriotism, and the more personal experience with guns they indicated. Including any of these variables as a covariate in the ANOVA on attitudes toward guns increased the effect size associated with condition, compared to the model without covariates. In other words, the difference in gun attitudes between the masculinity threat and the control condition was larger when any covariate was included, compared to not. As Table 4 shows, in six of the nine tested covariance models (3 covariates × 3 samples based on the different exclusion criteria), attitudes toward guns were significantly more positive after a masculinity threat than in the control condition.

**Table 3:** Means and standard deviations of all reported variables and pearson correlations among them in the full sample (*N* = 168).

Variable	*M* (*SD*)	1	2	3	4	5	6
1. Condition	–	–					
2. Gun attitude	3.27 (1.03)	0.17^*^	–				
3. Voucher^a^	–	0.15^*^	0.37^*^	–			
4. SDO	2.75 (1.64)	0.01	0.28^*^	0.17^*^	–		
5. Patriotism	4.11 (1.80)	−0.04	0.43^*^	0.10	0.26^*^	–	
6. Gun experience	3.30 (2.51)	0.06	0.41^*^	0.17^*^	0.18^*^	0.15^*^	–

Scales ranges were: Gun attitudes (GAS) 1–5; SDO 1–9; personal experience with guns 1–9. ^a^Taking a voucher was a dichotomous variable, with *n* = 153 because of missing data. *p < 0.05 (two-tailed).

**Table 4:** ANCOVA findings on the influence of covariates on the effect of condition on attitudes toward guns with different exclusion criteria (maximal, no-suspicion, and minimal sample).

Sample and effect of…	*F*	*p* ^a^	η_p_^2^
Maximal (*N* = 168; *df* 1, 165):			
Social dominance orientation	14.20	<0.001	0.08
Condition	4.73	0.014	0.03
Patriotism	33.98	<0.001	0.20
Condition	7.10	0.004	0.04
Experience with guns	40.26	<0.001	0.20
Condition	7.10	0.004	0.04
No-suspicion (*N* = 141; df 1, 138):			
Social dominance orientation	12.13	<0.001	0.08
Condition	2.73	0.051	0.02
Patriotism	32.03	<0.001	0.19
Condition	3.26	0.037	0.02
Experience with guns	26.13	<0.001	0.16
Condition	3.03	0.042	0.02
Minimal (*N* = 103; df 1, 100):			
Social dominance orientation	2.66	0.053	0.03
Condition	2.40	0.063	0.02
Patriotism	18.36	<0.001	0.16
Condition	2.18	0.072	0.02
Experience with guns	19.73	<0.001	0.17
Condition	3.12	0.029	0.04

^a^One-tailed *p*s are reported because all assumptions regarding relationships were directed.

## Discussion

The main aim of this study was to examine whether men are more attracted to guns after having been threatened with respect to their masculinity. Extending recent research by Borgogna et al. (2022), we conducted an in-person experiment with two novel dependent variables, general attitudes toward guns and, as a behavioral indicator, choice to accept a free voucher for shooting training at a gun range. Descriptively, results were as expected, yet whether findings were statistically significant or not depended on participant inclusion/exclusion decisions and the inclusion of covariates.

To our knowledge, this is the second experiment that found increased attraction to guns after a masculinity threat, after the recent publication by Borgogna et al. (2022). As those authors discuss, a controlled in-person setting leads to a higher internal validity, compared to their study. Such a setting was used in the present study which thus served as a conceptual replication and extension of that experiment. We also used a different masculinity threat (i.e., the gender knowledge test instead of false personality feedback), further extending the evidence base. With respect to causality, due to the experimental design any change with respect to one of the dependent variables theoretically should be caused only by the experimental manipulation. Thus, the two existing experimental tests of the relationship between masculinity threat and attraction to guns are highly valuable additions to the existing correlational studies (Mencken and Froese, 2019; Cassino and Besen-Cassino, 2020; McDermott et al., 2021; Scaptura and Boyle, 2022). Taken together, both experiments suggest that attraction to guns can be an overcompensatory reaction by men after a masculinity threat (Bosson and Vandello, 2011; Willer et al., 2013).

Referring to the included covariates (i.e., social dominance orientation, patriotism, and experience with guns), several of them led to an increase of the effect size regarding the experimental manipulation. Higher social dominance orientation, higher patriotism, and more experience with guns went along with more positive gun attitudes, as expected based on the literature (Pratto et al., 1994; Mencken and Froese, 2019; Shapira et al., 2022). Probably, these variables caused the corresponding changes by accounting for error variability. The theoretical relation between gun attitudes and gun experience is debatable: Both causal directions are plausible. On the one hand, gun attitudes should determine how much real-world gun experience people seek, and on the other, (negative) experiences with guns should affect gun attitudes (for discussion, see Shapira et al., 2022). In contrast, the other two variables, patriotism and especially social dominance orientation, are considered powerful predictors of a wide range of social, political, and intergroup attitudes and behavior. As replicated here, gun attitudes are included in this range. Our findings indicate that including important covariates makes experiments on masculinity threat and gun attitudes more powerful statistically.

Taken together, the available evidence in the present study and in that by Borgogna et al. (2022) suggests that there exists a small-to-medium size effect of masculinity threat on gun attitudes in the population. This is in line with our hypotheses.

### Implications of the present work

Precarious manhood theory (Bosson and Vandello, 2011) and the masculine overcompensation hypothesis (Willer et al., 2013) suggest that men try to confirm their status as men in reaction to situations that challenge their masculinity. Research to date has confirmed this hypothesis with regard to a large range of different reactions, some of them rather funny such as preferring masculine-associated over feminine-associated food and drinks (e.g., coffee “Joe” vs. “café latte,” Gal and Wilkie, 2010). However, many of men’s compensatory reactions that should prove their masculinity are instead harmful and have negative consequences for men themselves, for others, or for society as a whole. For example, after a masculinity threat, men overcompensated by expressing more toughness – setting a higher voltage they would be willing to endure (Fowler and Geers, 2017). Threatened men are also more willing to take risks, which can harm them (Parent et al., 2018). Examples of negative consequences for others are that heterosexual men have harassed an allegedly gay (Talley and Bettencourt, 2008) or a female interaction partner as a compensation (Maass et al., 2003) following a masculinity threat. Exemplifying negative consequences to society, men’s attitudes toward father roles and gender equality became more traditional after a masculinity threat (Kosakowska-Berezecka et al., 2016), and under economic threat, men tended to reject an equal distribution of resources and act less prosocially (White et al., 2013). The present findings extend the range of negative consequences of precarious manhood that are potentially harmful to society by demonstrating that men’s gun attitudes can be more negative after a masculinity threat (as compared to a masculinity affirmation). Thus, our research extends the evidence base regarding precarious manhood theory and the masculine overcompensation hypothesis.

The most fundamental aim with respect to “the United States gun problem” (Winker et al., 2016, p. 1) should be to generate as much knowledge as possible to understand the related phenomena in the best possible way. Hence, the most important implication of the present study is that it contributes to theory building with respect to the relationship between masculinity threats and attraction to guns. Thus, when we know more about reasons for men’s attraction to guns including mediating and moderating factors; when we understand what drives men to embrace these kinds of objects, then this knowledge can contribute to enduring changes in a society’s consciousness with respect to this topic, on a collective and on an individual level. Based on such knowledge, interventions can be developed. For example, regarding coaching and therapy, such knowledge can possibly contribute to new approaches fostering individual change. Regarding men’s need for feeling and being seen as strong, especially in the face of threats to their masculinity, therapeutic techniques could help men to find other ways of “finding strength” instead of compensating via more attraction to guns.

An implication for research is that the masculinity-threat effect on gun attitudes and attraction that we reported was smaller than expected *a priori*. Future research should thus use larger samples and plan to include covariates, both to find more robust effects and to examine moderating factors.

### Limitations

Sample size can be regarded a first limitation of our study. Borgogna et al. (2022) found statistically significant effects, with effect sizes around *d* = 0.35 (i.e., smaller than we expected in our *a-priori* power analysis). This could help explain why our effects were statistically significant in many, but not all, statistical analyses. Additionally, it is possible that Borgogna et al.’s (2022) dependent variable is more sensitive to a masculinity threat compared to the dependent variables used in the present study. To measure attraction to guns, they presented participants with four different pictures of firearms and asked them about their interest in owning each one. In contrast, the general attitudes toward guns that we measured could be based, at least to a certain extent, on an individual history of personal experiences with guns (e.g., Loyd and Gressard, 1984). Hence, the attitudes we measured could be more stable and thus, more difficult to change by a masculinity threat, compared to a momentary evaluation of specific guns. Thus, the inclusion of covariates might be necessary to detect these smaller effects in our study. Our robustness checks support this reasoning.

As a second limitation, since the recruitment procedures did not allow us to obtain a nationally representative U.S. sample, the results of the present study cannot be generalized to all American men. The majority of participants were young White men who came from suburban communities in Florida and did not own guns personally. We used different recruitment strategies. On the one hand this can be regarded a strength in that not all participants were college students, thus the sample’s representativeness is increased over a mere student sample. But on the other hand, it is a weakness because characteristics of participants differ, depending on recruitment strategy, which may induce bias. Together with the fact that our participants were all from Florida, United States, the external validity of our experiment is limited in this regard.

Another issue worth discussing is the suspicion check we included. In short, we found it impossible to present an algorithm-like decision tree that would allow us to double-check suspicion ratings and compute interrater reliability. The research assistants were trained with respect to the suspicion check and additionally, reflection and feedback sessions were performed with each of them. However, it seems that they still used different criteria for determining suspicion. As the presented analyses show (see Table 1), the implementation of the suspicion check at least did not bias the results in a way that led to false positive results because the hypothesized effect became non-significant in the no-suspicion sample. Apparently, this was not simply due to the reduced sample size because in a third, minimal sample in which all possible low-quality data were excluded, the effect size increased again.

In hindsight, it is possible that voicing suspicion itself could be a compensatory reaction. If a man receives feedback that threatens his masculinity, one possible “defense reaction” is publicly disavowing the feedback (e.g., “this knowledge test is not valid for assessing real masculinity”; see, e.g., Greve and Wentura, 2003, for related reasoning). Thus, higher suspicion may actually be an indicator of higher masculinity threat. Excluding suspicious participants may thus threaten, rather than protect, internal validity.

There appears to be another way in which false feedback may threaten the validity of masculinity-threat experiments. In an online replication study with experienced Prolific participants (*N* = 580), we found no statistically significant effects. We learned afterwards that the majority of participants had previously taken part in more than 1,000 studies, most others in more than 500, the remaining ones at least in more than 100. We thus suspect that the manipulation in that experiment failed because those participants, experienced with deception, did not believe the false feedback they received regarding their masculinity; thus, no threat occurred. For full transparency these data are available in the Online Supplementary Materials accompanying the present paper. A clear implication for future research is not to recruit experienced online samples for testing experimental hypotheses that include relatively blatant false (masculinity-threatening) feedback.

### Future research directions

Future studies should examine the influence of different kinds of masculinity threats on participants’ attraction to guns. The experimental manipulation used in the present study (i.e., lack of masculine knowledge) as well as the one used by Borgogna et al. (2022) (i.e., lack of masculine personality) consisted of a false masculinity threatening feedback. It is possible that for highly masculine men, it is easy not to believe the masculinity threatening information. To increase the effects of this kind of threat, researchers could heighten the publicness of the feedback by, for example, including an audience. This could especially threaten men adhering strongly to an honor ideology (Stratmoen et al., 2018). Moreover, other kinds of masculinity threats could be used to find out more about similarities and differences between them with respect to their effects on attraction to guns. It is possible, for example, that compensation is most effective if it occurs in the same domain as the threat. That is, perhaps threats to one’s masculine knowledge are better compensated for by demonstrations of one’s masculine knowledge than by demonstrations in other domains, such as physical strength. A further example of a masculinity threat would consist of a situation in which male participants lose in any kind of masculinity-related competition against a female interaction partner, ideally during a personal confrontation. Such studies would yield evidence on the specificity versus generality of masculinity-threat compensation and thus indicate which interventions could be used: Can masculinity be restored by less dangerous means than attraction to guns and shooting?

Additionally, it is desirable to know whether our theoretical reasoning generalizes to societies other than the United States. Indeed, the firearm culture in the United States seems to be relatively unique compared to other countries (Carson et al., 2022) which probably has a particular influence on United States men compared to men from other countries. However, it is possible that similar mechanisms apply to men in other Western cultures, or that they generally apply to all men, due to similar fundamental socio-cultural and psychological mechanisms of humans (but see Valved et al., 2021, for cultural differences in masculinity threat). Future research should thus test the influence of masculinity threat on attraction to guns in other cultures.

Also, attraction to guns could be operationalized in different ways. If the effects replicated across several different operationalizations, this would increase confidence in the hypothesis that masculinity threats increase men’s attraction to guns. Additionally, this would offer the possibility to find out more about the real-world impact of masculinity threats. For example, if a masculinity threat led to more gun purchases among threatened men this would be an especially compelling indicator. Hence, more directly behavior-related dependent variables should be used. Our shooting-range voucher was a first step in this direction.

Moreover, further potential moderator variables could be tested, for example men’s general self-esteem or, more specifically, “masculinity contingency” which is defined as “the degree to which a man’s self-worth is derived from his sense of masculinity” (Burkley et al., 2016, p. 113). This would broaden our knowledge about characteristics of men facilitating the compensation of a threat to their masculinity via a stronger attraction to guns. The more knowledge can be generated about this topic, the better we can understand the mechanisms between threatened masculinity and attraction to guns.

In the theory section we argued that men could be more attracted to guns than women because the social status of being a man is more insecure and instable than the social status as a woman and that as a consequence men have to confirm and reaffirm their masculine status permanently and especially when it is threatened directly (Vandello and Bosson, 2013). Since being strong seems to be an important feature of “a real man,” one way to compensate for masculinity threats can consist of any demonstration of strength, which guns may provide (Fessler et al., 2012). Yet, while this theoretical reasoning reflects one plausible line of argument it is not the only possible answer to the basic question why men are more attracted to guns than women.

## Conclusion

Guns cause serious problems in families and society. Could men’s attraction to guns be based on precarious manhood, such that men who feel threatened in their masculinity as a compensation report more positive attitudes toward guns and feel more attracted to practice shooting? The findings of the present experiment suggest that this could be the case (replicating and extending recent findings by Borgogna et al., 2022). After receiving the feedback that they lacked typically masculine knowledge, men showed more positive attitudes toward guns and were more likely to choose a gun-range voucher than men in the control condition. The present work thus extends evidence on the negative consequences for society that can result from masculinity threats. Hence, it contributes to knowledge about the relationship between masculinity threats and guns, and in the long run, this could change how societies deal with firearms and thus contribute to a more humane social coexistence.

## Data availability statement

The datasets presented in this study can be found in online repositories. The names of the repository/repositories and accession number(s) can be found in the article/supplementary material.

## Ethics statement

The studies involving humans were approved by Institutional Review Board of the University of South Florida. The studies were conducted in accordance with the local legislation and institutional requirements. The participants provided their written informed consent to participate in this study.

## Author contributions

SK: Conceptualization, Funding acquisition, Supervision, Writing – review & editing. TB: Conceptualization, Formal analysis, Methodology, Project administration, Writing – original draft. JB: Conceptualization, Methodology, Supervision, Writing – review & editing. LL: Writing – review & editing. MS: Conceptualization, Formal analysis, Funding acquisition, Methodology, Supervision, Writing – original draft.

## Footnotes

^1^ https://researchbox.org/1902

^2^ https://www.qualtrics.com
